# Identifying indispensable proteins of the type III secretion systems of *Salmonella enterica* serovar Typhimurium strain LT2

**DOI:** 10.1186/1471-2105-13-S12-A10

**Published:** 2012-07-31

**Authors:** Chandrajit Lahiri, Pawar Shrikant, Radhakrishnan Sabarinathan, M Izhar Ashraf, Dipshikha Chakravortty

**Affiliations:** 1 Center for Infectious Diseases Research, Department of Microbiology and Cell Biology, Indian Institute of Science, Bangalore 560012, India; 2 Department of Bioinformatics, School of Biotechnology and Health Sciences, Karunya University, Coimbatore 641114, India; 3 Department of Biology, Western Kentucky University, Bowling Green, Kentucky 42101, USA; 4 Department of Cell and Developmental Biology, Cornell University, Weill Cornell Medical College, Manhattan, NY 10065, USA; 5 Bioinformatics Center, Indian Institute of Science, Bangalore - 560012, India; 6 Theoretical Physics, The Institute of Mathematical Sciences, Chennai - 600113, India

## Background

An estimated 1.3 billion cases of salmonellosis occur worldwide every year [1]. The causative organism *Salmonella enterica* serovar Typhimurium strain LT2 uses different proteins to inject into the host cells to cause such systemic infection. Several reports exist on the role and importance of these individual proteins of the Type III secretion system of *Salmonella* pathogenicity islands (SPI). Two component signal transduction system plays a major role in regulation of those virulent SPI genes [2]. However, the most indispensable of them and their hierarchical role has not been worked out in detail.

## Materials and methods

We have adopted a graph theoretical approach to build a network of these and other associated signal transduction proteins and utilized modules like centrality measures and network decomposition to analyze our result. An initial approach to get the fingerprint of the network via k-core analyses listed out the set of proteins InvA, InvE, InvG, InvF, PrgK, SicA, SipA, SipB, SipC, SpaK, SpaL, SpaO, SpaP, SpaQ, SpaR, SpaS, SsaJ, SsaK, SsaL, SsaM, SsaN, SsaO, SsaP, SsaQ, SsaS, SsaT, SsaU, SsaV, and YscR which comes into action in the process of invasion and colonization and thus become indispensable than the rest. All the significant proteins identified were confirmed by Agilent Microarray with subsequent Cytoscape analysis.

## Results

The chaperone protein SicA was figured out to be the most indispensable one from classical centrality measures and confirmed by microarray analyses as well. We also propose a hierarchy of the proteins involved in the total infection process. Our method is the first of its kind to figure out, albeit theoretically, potential virulence determinants encoded by SPI for therapeutic targets for enteric infection.

## Conclusions

Target genes were identified and then validated by using independent, published microarray data. The result is a targeted set of genes that are sensitive predictors which could then form the basis for a series of tests in the wet-lab background. Understanding these regulatory and virulent genes will provide insight into conditions which are encountered by this intracellular enteric pathogen during the course of infection which will further contribute in identifying new targets for antimicrobial agents.
